# BCL2A1a Over-Expression in Murine Hematopoietic Stem and Progenitor Cells Decreases Apoptosis and Results in Hematopoietic Transformation

**DOI:** 10.1371/journal.pone.0048267

**Published:** 2012-10-30

**Authors:** Jean-Yves Métais, Thomas Winkler, Julia T. Geyer, Rodrigo T. Calado, Peter D. Aplan, Michael A. Eckhaus, Cynthia E. Dunbar

**Affiliations:** 1 Hematology Branch, National Heart, Lung, and Blood Institute, National Institutes of Health, Bethesda, Maryland, United States of America; 2 Department of Pathology and Laboratory Medicine, Weill Cornell Medical College, New York, New York, United States of America; 3 Department of Internal Medicine, University of São Paulo at Ribeirão Preto Medical School, Ribeirão Preto, Brazil; 4 Genetics Branch, National Cancer Institute, National Institutes of Health, Bethesda, Maryland, United States of America; 5 Division of Veterinary Resources, National Institutes of Health, Bethesda, Maryland, United States of America; Emory University, United States of America

## Abstract

We previously reported the development of a lethal myeloid sarcoma in a non-human primate model utilizing retroviral vectors to genetically modify hematopoietic stem and progenitor cells. This leukemia was characterized by insertion of the vector provirus into the *BCL2A1* gene, with resultant BCL2A1 over-expression. There is little information on the role of this anti-apoptotic member of the BCL2 family in hematopoiesis or leukemia induction. Therefore we studied the impact of Bcl2a1a lentiviral over-expression on murine hematopoietic stem and progenitor cells. We demonstrated the anti-apoptotic function of this protein in hematopoietic cells, but did not detect any impact of Bcl2a1a on in vitro cell growth or cell cycle kinetics. *In vivo,* we showed a higher propensity of HSCs over-expressing Bcl2a1a to engraft and contribute to hematopoiesis. Mice over-expressing Bcl2a1a in the hematologic compartment eventually developed an aggressive malignant disease characterized as a leukemia/lymphoma of B-cell origin. Secondary transplants carried out to investigate the primitive origin of the disease revealed the leukemia was transplantable. Thus, Bcl2a1 should be considered as a proto-oncogene with a potential role in both lymphoid and myeloid leukemogenesis, and a concerning site for insertional activation by integrating retroviral vectors utilized in hematopoietic stem cell gene therapy.

## Introduction

Recently we reported the development of an acute myeloid leukemia in a rhesus macaque transplanted with autologous CD34+ cells transduced with a murine stem cell virus-derived replication defective retroviral vector expressing only marker genes under control of the strong MCSV long terminal repeat (LTR). This animal had an unusual clonal reconstitution pattern the first year following transplant, with a single transduced myeloid progenitor cell clone accounting for up to 80% of the then normal myelopoiesis [1]. The same vector-containing clone eventually transformed to AML five years following transplantation, and each tumor cell was shown to contain two vector insertions, one localized 20 kb upstream of the CDw92 gene on chromosome 9, and the second localized in the first intron of BCL2A1 on chromosome 15 [2], a gene belonging to the anti-apoptotic BCL2 family not previously linked to myeloid leukemia. BCL2A1 was highly expressed in the tumor cells. This tumor was the first hematopoietic malignancy reported in a recipient of primitive cells transduced with a replication-incompetent vector containing only marker genes, and suggested that BCL2A1 could have potent effects on hematopoiesis.

BCL2A1, also known as Bfl-1, GRS, or BCL2L5, belongs to the BCL2 family of anti-apoptotic proteins. Murine BCL2A1 was originally identified as a gene product induced by the stimulation of primary bone marrow cells with GM-CSF [3]. The human homolog was later cloned in three independent studies [4]–[6]. Initially its expression was thought to be specific to the hematopoietic compartment [7], but further studies showed a less restrictive expression pattern [5]. For instance, expression of Bcl2a1 starts at day E11.5 in brain, limbs, and liver during mouse embryogenesis. At day E15.5 it is also detected in yolk sac, heart, thymus, lung, kidney, and spleen [8]. In mice the *Bcl2a1* gene is duplicated, giving rise to 4 variants named *Bcl2a1a, b, c*, and *d* [9]. This phenomenon is not shared by other species even though 2 splice variants with the same function were described in humans [10].

*Bcl2a1a* knock-out in C57BL/6 mice is not lethal, and mice have a normal lifespan. However, neutrophils of *Bcl2a1a*-deficient mice are more prone to apoptosis, suggesting an anti-apoptotic function for this protein [11]. A recent study used RNAi to down-regulate all of the murine isoforms of *Bcl2a1*, and demonstrated diverse actions of this gene product in the development and homeostasis of T cells, B cells and granulocytes [12]. During normal myeloid maturation, neutrophilic differentiation is linked to up-regulation of BCL2A1, in part via activation of the BCL2A1 promoter by the transcription factor PU.1 [13]. In myeloid leukemias, PU.1 and BCL2A1 expression are highly correlated [13]. Expression of BCL2A1 can be induced by various molecules such as tumor necrosis factor α (TNFα), phorbol 12-myristate 13-acetate (PMA), lipopolysaccharide (LPS), or interleukin-1 (IL-1) (e.g.: [14], [15], [5]). NFKB directly regulates the expression of BCL2A1 and recruits a large transcriptional complex at its promoter [16]–[18]. Most anti-apoptotic proteins, when over-expressed or deregulated, act to induce or accelerate hematologic malignancies, often by potently interacting with mutations in proliferative pathways such as deregulating of MYC, or interfering with p53 death signals [19]–[21]. The first member of this class, *BCL2*, was discovered as the gene activated by a chromosomal translocation in virtually all cases of human follicular lymphoma [22].

There is limited information linking BCL2A1 expression and human hematologic malignancies. In one small study *BCL2A1* mRNA was over-expressed in acute lymphoid leukemia (ALL), acute myeloid leukemia (AML), chronic lymphoid leukemia (CLL), chronic myeloid leukemia (CML), and mantle cell lymphoma tumor cells compared to normal marrow cells [23]. Levels were particularly elevated in AML patients with a normal karyotype. However, the potential role of BCL2A1 as a marker for subtypes of AML needs further study [23]. In a murine model, similar to other anti-apoptotic proteins, *BCL2A1* expression has recently been shown to accelerate the onset of myeloid leukemia induced by MYC over-expression. Other anti-apoptotic BCL2-family genes were also tested (*BCL2, BCLxL, BCLw, MCL1,* and *BCLb*) in this model, and *BCL2A1* was the least potent cooperative gene with MYC in AML induction [24]. However, the impact of *BCL2A1* expression alone was not studied in this set of experiments.

In this study we aimed to better understand the potential role of dysregulation of the *Bcl2a1a* gene in etiology of leukemia. We hypothesized that over-expression of *Bcl2a1a* would confer a selective advantage to the cells through its anti-apoptotic function, facilitating eventual development of leukemia. Hence, we over-expressed *Bcl2a1a* in the hematologic compartment in a murine model, in order to study its impact on hematopoiesis and the possible emergence of malignant disease.

## Materials and Methods

### Ethics Statement and Animal Care

All mice were housed and handled in strict accordance with the recommendations in the “Guide for the Care and Use of Laboratory Animals” of the National Institutes of Health, Bethesda, MD. All animal experiments were carried out on National Heart Lung and Blood Institute Animal Care and Use Committee–approved protocol number H-1031R2.

### Vector Construction and Production

**Figure 1:**
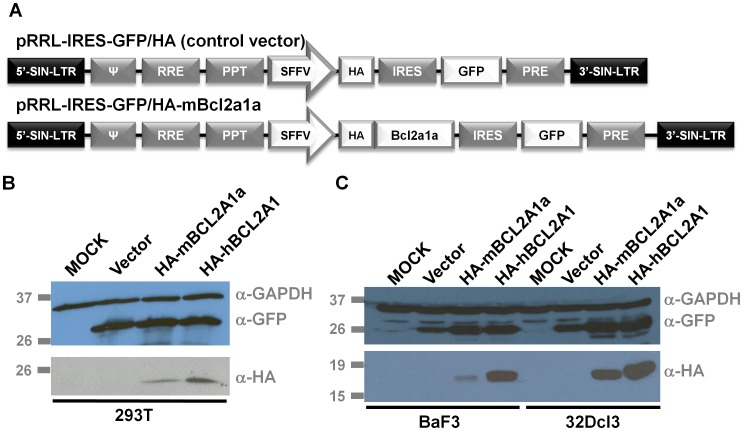
Construction of BCL2A1 lentiviral vectors, and demonstration of high level expression in producer and transduced cell line.(A) Schematic of the lentiviral vectors used for expression of murine BCL2A1a or human BCL2A1. LTRs are self inactivating (SIN), ψ (packaging sequence), RRE (Rev-Responsive Element), PPT (Polypurine Tract), SFFV (Spleen Focus-Forming Virus promoter), IRES (Internal Ribosome Entry Site), HA (Hemagglutinine antigen), GFP (Green Fluorescent protein), PRE (post-transcriptional regulatory element). (B,C) Expression was assessed by western blot using an anti-HA antibody to recognize HA-tagged BCL2A1, or anti-GFP, and anti GAPDH as a loading control. Western blots were carried out in 293T producer cell lines (B) and in transduced BAF3 and 32Dcl3 cells (C).

Murine *Bcl2a1a* cDNA was cloned from total RNA extracted from C57BL/6 mouse heart using the Qiagen RNEasy mini kit (Qiagen, Hilden, Germany), following first strand cDNA synthesis via the Superscript First Strand kit (Invitrogen™, Life Technologies, Carlsbad, CA). Primers mBCL2A1AFOR(1-21) 5′-ATGGCTGAGTCTGAGCTCATG-3′ and mBCL2A1AREV(519-498) 5′-TTACTTGAGGAGAAAGAGCAT-3′ were used to amplify the open reading frame of the gene and the PCR product was subcloned into the pCR4TOPO vector (Invitrogen™, Life Technologies). The human *BCL2A1* gene was purchased from B-Bridge International (Clone IRATp970D1012D, Cupertino, CA). After amplification by PCR with primers introducing a Kozak sequence as well as restriction enzyme sites, the genes were subcloned into the pMIEV-IRES-GFP retroviral vector. An HA tag was also added to pMIEV-IRES-GFP vectors with the Stratagene Quick Change II site directed mutagenesis kit (Agilent Technologies, La Jolla, CA). The HA-tagged murine *Bcl2a1a* and human *BCL2A1* sequences were introduced into the pRRL.PPT.SF.GFPpre lentiviral vector along with the IRES sequence [25]. Vectors utilized in these studies are summarized in Figure 1A.

To produce lentiviral particles, 293T cells were seeded at 5 million cells per 10-centimeter dish. Twenty-four hours later, cells were co-transfected (Calcium Phosphate Transfection Kit, Sigma-Aldrich, Saint Louis, MO) with the following plasmids: pCDNA3.HIVgag/pol.4xCTE (12 ug), pMD2.G-VSV-G (1 ug), pRSV-REV (5 ug), and 10 ug of pRRL.PPT.SF.IRES.GFPpre/HA, pRRL.PPT.SF.IRES.GFPpre/HA-mBcl2a1a, or pRRL.PPT.SF.IRES.GFPpre/HA-hBCL2A1. Vector-supernatants were collected once a day for 3 days, filtered through 0.22-um filters, and concentrated by ultracentrifugation for 2 hr at 71 900 g and 4°C in a SW28 rotor (Beckman Coulter, Atlanta GA). Pelleted viral particles were resuspended in 1 mL of StemSpan® (StemCell Technologies Inc., Vancouver, Canada) media and stored at minus 80°C. Viral titers were determined on NIH 3T3 cells via standard assays [26].

### Cell Culture and Transduction

The murine myelomonocytic WEHI-3 (ATCC, #TIB-68, Manassas, VA) cell line was grown in IMDM supplemented with 10% FCS, 2.5 mM β2-mercapto-ethanol, glutamine, penicillin and streptomycin. Murine NIH3T3 (ATCC, #CRL-1658) and human 293T (ATCC, #CRL-11268) fibroblastic cell lines were grown in I10 media (IMDM supplemented with 10% FCS, glutamine, penicillin and streptomycin). The murine pro-B BaF3 (Leibniz-Institut DSMZ, #ACC300, Braunschweig, Germany) and murine myeloblast-like 32Dcl3 (ATCC, #CRL-11346) cell lines were grown in RPMI1640X supplemented with 10% WEHI-3 conditioned media as a source of IL-3, 10% fetal calf serum (FCS Heat-inactivated, Sigma-Aldrich), glutamine, penicillin and streptomycin (Gibco, Life Technologies, Grand Island, NY). The human erythroblastoid UT7/Epo-S1 cell line was grown in I10 media containing 2 U/mL of human erythropoietin (Amgen, Thousand Oaks, CA). Fifty thousand BaF3, 32Dcl3, or UT7/Epo-S1 cells [27] were transduced with vector particles on retronectin-coated plates (Takara, Madison, WI) at a multiplicity of infection (MOI) of 5 in presence of protamine sulfate 4 ug/mL (Sigma-Aldrich).

Murine bone marrow cells were obtained by flushing bone marrow from humeri, femurs, and tibias of C57BL/6 Ly5.1 mice (C57BL/6 Ly5.1 (Pep3b) Stock 002014, Jackson Laboratories, Bar Harbor, ME). Red cells were lysed with ACK buffer (Quality Biological Inc., Gaithersburg, MD). Nucleated cells were purified using the MACS mouse lineage depletion kit (Miltenyi Biotec, Auburn, CA), and the resulting lineage negative progenitor cells were stimulated for two days at a starting density of 5×10^5^ cells/mL in StemSpan® media supplemented with 10 ng/mL murine IL-3 (R&D Systems, Minneapolis, MN), 100 ng/mL murine IL-11 (R&D Systems), 50 ng/mL murine stem cell factor (Research Diagnostic Inc., Concord, MA), and 100 ng/mL human Flt-3 Ligand (R&D Systems). Each recipient mouse was transplanted with the equivalent of 5×10^5^ stimulated cells that were transduced twice with lentiviral supernatants at a MOI of 5 on retronectin-coated plates in the presence of protamine sulfate 4 ug/mL.

### Murine Transplantation and Follow-up

Transduced lineage negative bone marrow cells were resuspended in StemSpan® media. Following 900 rads total body irradiation each recipient C57BL/6 Ly5.2 mouse (Stock 000664, Jackson Laboratories) received transduced cells resuspended in 500 uL media via tail vein injection.

To perform secondary transplants, bone marrow cells were collected by flushing humeri, femurs, and tibias of primary mice, and red cells were lysed using ACK buffer. Using a ratio of 1 primary mouse for 3 secondary mice, primary bone marrow cells were reinfused by tail vein injection into sub-lethally irradiated secondary mice C57BL/6 (Ly5.2) (900 rads total body irradiation).

Blood was collected monthly via retro-orbital bleeding, and used for a complete blood count (Hemavet 950FS, Drew Scientific, Waterbury, CT), blood smear (hematoxylin and eosin (H&E) stained), and flow cytometric analysis.

Organs including heart, lung, kidney, spleen, liver, salivary glands, lymph nodes, any obvious tumor masses, and sternum from pre-morbid mice were collected, fixed in 10% formalin (Fischer Scientific, Thermo Fisher Scientific Inc., Pittsburgh, PA), and embedded into paraffin blocks for subsequent sectioning and H&E staining (Histosev, Gaithersburg, MD) or immunohistochemistry. Single cell bone marrow, lymph node, and spleen suspensions were obtained by flushing tissues with media. Cytospins were prepared from 10^5^ cells, using 300 rpm for 5 minutes (CytoSpin 4 Cytocentrifuge, Thermo Scientific, Thermo Fisher Scientific Inc.). Cell suspensions were analyzed by flow cytometry. Genomic DNA was isolated from cells or tissues using Qiagen DNeasy Tissue and Blood kit according to manufacturer’s recommendation.

### Flow Cytometric Analysis and Cell Sorting

Transduced BaF3, 32Dcl3, and UT7/Epo-S1 cells were sorted for GFP expression using a BD FACSAria cell sorter from BD Bioscience (San Jose, CA). Peripheral blood, bone marrow, and spleen cells were resuspended in FACS buffer (2.7 mM Potassium chloride, 1.5 mM Sodium phosphate dibasic heptahydrate, 1.5 mM Potassium phosphate monobasic, Sodium chloride 137 mM, Sodium azide 7.7 mM, 1% (w/v) BSA in water) and incubated with the following cocktail of antibodies from BD Pharmingen™ (BD Bioscience, San Diego, CA): T-cells-anti-mouse CD3e (Hamster, APC), granulocytes and NK cells-anti-mouse CD11b (Rat, PE-Cy7), B-cells-anti-mouse CD45R/B220 (Rat, APC-Cy7). Engraftment of donor cells was monitored via anti-mouse CD45.1 (Mouse, R-PE) and anti-mouse CD45.2 (Mouse, PerCP-Cy5.5). After washing with FACS buffer, cells were analyzed with a BD™ LsrII flow cytometry system from BD Bioscience. Data were analyzed using FlowJo from Treestar Inc., Ashland, OR).

### Cell Cycle and Apoptosis Assays

Cell cycle status was assessed using the NuCycl™ PI Kit from Exalpha (Watertown, MA) according to the manufacturer’s recommendation. Apoptosis was assessed using the Annexin V-PE apoptosis detection kit I from (BD Pharmingen™, BD Biosciences). Analysis of cell cycle and apoptosis was performed on a BD™ LsrII flow cytometer. Data were processed using ModFit™ software from Verity Software House (Topsham, ME).

### Immunohistochemistry

Immunohistochemical staining was performed on formalin-fixed, paraffin-embedded tissue sections from the spleen, liver, sternal bone marrow, affected lymph nodes, and tumor masses. Cut tissue sections were deparaffinized, and endogenous peroxidase was inactivated. Antibodies included rat anti-mouse B220 (clone RA3-6B2, BD Pharmingen™, BD Biosciences), rabbit polyclonal anti-mouse CD3 (Dako, Glostrup, Denmark), rat anti-mouse CD34 (Clone MEC 14.7, Abcam, Cambridge, MA), rat anti-mouse CD68 (Clone FA-11, Abcam), mouse anti-mouse Lysozyme (clone BGN/06/961, Abcam), rat anti-mouse Mac2 (Clone M3/38, Cedarlane Laboratories Limited, Burlington, NC), rabbit anti-human myeloperoxidase (MPO) with cross-reactivity to mouse (Dako), peanut agglutinin (Vector Laboratories, Burlingame, CA), and rabbit anti-human TdT with cross-reactivity to mouse (Supertechs Inc., Rockville MD). For CD3, CD34, lysozyme, Mac2 and MPO staining, antigen retrieval was performed using either the Bond Epitope Retrieval Solution 1 (ER1) or the Bond Epitope Retrieval Solution 2 (ER2) (Leica Biosystems, Newcastle Upon Tyne, UK) at 99–100°C for 20–30 min. Enzymatic retrieval was performed for CD68. TdT was kept in a pressure cooker in citrate buffer with pH = 6 for 10 minutes. Subsequently, CD3, MPO, and TdT sections were incubated with the primary antibody for 30 minutes, followed by incubation with enVision + System HRP labeled polymer anti-rabbit secondary antibody (Dako) for 25 minutes. Antibodies for B220, CD34 and Mac2 were incubated with the primary antibody for 30 minutes, followed by a secondary biotinylated polyclonal goat anti-rat antibody (BD Pharmingen™, BD Biosciences) for 25 minutes and Streptavidin-HRP (Dako) for 25 minutes. Lysozyme was stained using the Animal Research Kit Peroxidase for mouse primary antibodies (Dako), as described by the manufacturer. Sections underwent colorimetric development with diaminobenzidine (DAB, Leica Biosystems), were counterstained with hematoxylin, dehydrated using graded alcohols and mounted in Cytoseal™ XYL (Richard-Allan Scientific, Kalamazoo, MI).

### Western Blot

Frozen spleen fragments were ground in a total volume of 750 uL of RIPA buffer (Sigma-Aldrich) supplemented with a cocktail of protease inhibitors (Complete Mini EDTA-Free and PhosSTOP, Roche, Indianapolis, IN). After clarification by centrifugation for 20 min at 20 800 g and 4°C, protein concentration was determined using the Protein Assay reagent (Bio-Rad, Hercules, CA). Proteins from 14 ug of lysate were separated by electrophoresis on 4–15% acrylamide Ready gel (Biorad) in Tris/Glycine/SDS buffer (Bio-Rad). Protein standards BenchMark™ and MagicMark (Invitrogen™, Life Technologies) were used as recommended by manufacturer. Proteins were transferred to nitrocellulose membranes (Nitrocellulose Membrane Filter Paper Sandwich, Invitrogen™, Life Technologies) for 1 hr at 90 V in 10% methanol Tris/Glycine buffer (Bio-Rad). Membranes were rinsed in water, and blocked in 5% milk (Bio-Rad)/0.05% Tween20 (Bio-Rad)/PBS, pH 7.4 for 1 hr at room temperature. Primary mouse monoclonal antibodies including anti-HA (Clone 16B12, Covance, Berkeley, CA), anti-GAPDH (Clone 6C5, Ambion, Austin, TX), or anti-GFP (Clone 7.1 and 13.1, Roche) were diluted in 5% milk/0.05% Tween/PBS and incubated at 4°C overnight. Membranes were washed twice in 0.05% Tween/PBS for 10 min at room temperature. Secondary anti-mouse immunoglobulins/HRP polyclonal antibody (Dako) conjugated to HRP was diluted in 5% milk/0.05% Tween/PBS and incubated at room temperature for 45 min. After washing three times with 0.05% Tween/PBS and once with PBS, the membrane was incubated with electrochemiluminescent substrate (LumiGLO^R^, Cell Signaling Technology, Danvers, MA). Membranes were then exposed to a Luminescent Image Analyser (LAS-400, Fujifilm, Tokyo, Japan).

### Real-time PCR

Total RNA was isolated using RNeasy mini kit according to manufacturer’s instruction (Qiagen). DNAse I (RNAse-free DNAse set, Qiagen) was added to remove any residual contaminating genomic DNA. Real-time PCR was carried out on 75 ng of total RNA per sample using the TaqMan® One-Step RT-PCR Master Mix Reagents Kit (Applied Biosystems, Foster City, CA) according to manufacturer’s instruction. Primers and probes designed to detect total murine *Bcl2a1a* and *β2-Microglobulin* were as follows: FOR-mβ2-Microglobulin 5′-CTTCAGCAAGGACTGGTCTTTC-3′; REV-mβ2-Microglobulin 5′-CGGCCATACTGGCATGCTTAAC-3′; Probe-mβ2-Microglobulin 5′FAM-TGAATTCACCCCCACTGAGACTG-3′TAMRA; FOR-mBCL2A1a 5′-GATACGGCAGAATGGAGGTT-3′; REV-mBCL2A1a 5′-GCATTTCCCAGATCTGTCCT-3′; Probe-mBCL2A1a 5′FAM-TGAACCCAAATCTGGCTGGCTG-3′TAMRA. Reactions were carried out in triplicates for each sample on an ABI PRISM 7900HT Sequence Detection System from Applied Biosystems. Copy numbers were calculated based on standard curves using plasmids harboring *Bcl2a1a* and *β2-Microglobulin* genes.

### PCRs for Immunoglobulin Variable Heavy Chain Rearrangements

An adaptation of the PCR-based method reported by Schlissel and colleagues was used [28], with MuO, V_H_558, V_H_7183, D_H_L, and D_H_R as forward primers and either J3 or J4 as reverse primers. PCRs were performed with 100 ng of genomic DNA, 16 uL of Platinum PCR SuperMix High Fidelity (Invitrogen™, Life Technologies), and 1 uL of each forward and reverse primer resuspended at 10 uM. We performed an initial denaturation step of 4 min at 94°C followed by 32 cycles of 1 min at 94°C, 1 min at 60°C, 1.75 min at 72°C, to end by a final extension of 10 min at 72°C. PCRs were loaded on 2% agarose gel in tris-acetate buffer.

### Southern Blot

Genomic DNA was digested overnight with HindIII/Sac1 (TCR) or PciI (GFP) and separated on a 0.8% agarose (Ultrapure Agarose, Invitrogen™, Life Technologies) gel and transferred overnight to a nylon membrane (Amersham Hybond™-XL, GE Healthcare, Buckinghamshire, UK). The TCRβ probe was obtained by digesting pBR322/TCRβ2 by BglII and the GFP probe was obtained by PCR performed on pRRL.PPT.SF.IRES.GFPpre/HA plasmid with SB-GFP-FOR 5′-GGCCACAAGTTCAGCGTGTC-3′ and SB-GFP-REV 5′-AGCTCGTCCATGCCGAGAGT-3′ primers. Probes were radiolabeled using a labeling Kit (Amersham Ready-To-Go DNA Labeling Beads (-dCTP), GE Healthcare) with radioactive phosphate (EasyTides Deoxycytidine 5′ triphosphate [α32-P], Perkin Elmer, Boston, MA).

### Statistical Analyses

Kaplan–Meier survival curves (overall survival and disease-free survival) were established using Prism 4 from GraphPad Software (La Jolla, CA). This software was also used for other statistical analyses. One-way ANOVA was used to compare more than 2 data sets. Two-way ANOVA was used to compare more than 2 data sets over time. Student t-test was used to compare 2 data sets.

## Results

### BCL2A1 is Efficiently Over-expressed in Transduced Cells

We chose to utilize lentiviral vectors to over-express BCL2A1, given their more robust expression levels, higher titers, and marked decrease in insertional mutagenesis as compared to standard retrovirus vectors [29]. Vector particles pseudotyped with the VSV-G envelope were produced via transient transfection of 293T cells, concentrated, titered and used to transduce several cytokine-dependent hematopoietic cell lines, including BaF3, 32DCl3, and UT7/Epo-S1. We documented expression of HA-tagged murine BCL2A1a and human BCL2A1 in both 293T producer cells and in transduced BaF3 and 32DCl3 cells by western blot, as shown in Figure 1B and 1C.

### BCL2A1 is Anti-apoptotic in Hematopoietic Cells but does not have an Impact on Cell Growth or Cell Cycle

**Figure 2:**
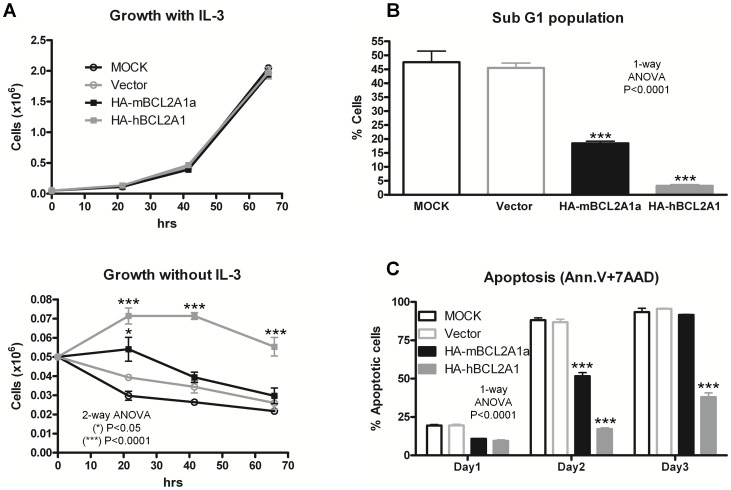
Impact of BCL2A1 over-expression in BAF3 cells. (A) Growth curves for BAF3 cells grown with or without murine IL-3. Viable cell numbers are shown over time. (B) Cell cycle analysis on BAF3 cells grown without IL-3. (C) Apoptosis assessed via Annexin V-PE staining in the absence of IL-3. All experiments were repeated 3 times and carried out in triplicates. Figure shows results obtained for one representative experiment. Data averages plus or minus standard deviations were plotted. HA-mBCL2A1a and HA-hBCL2A1 are respectively murine and human HA-BCL2A1.

BaF3 and 32Dcl3 cells are dependent on IL-3, and UT7/Epo-S1 cells are dependent on erythropoietin. When these cells are deprived of cytokines they undergo rapid apoptosis. The over-expression of murine HA-BCL2A1a or human HA-BCL2A1 did not alter the growth of BaF3 cells in the presence of IL-3 (Figure 2A). However, when deprived of IL-3, BaF3 cells over-expressing murine HA-BCL2A1a or human HA-BCL2A1 remained significantly more viable compared to cells transduced with a vector expressing GFP alone or control untransduced cells (MOCK) (Figure 2A).

We assessed the cell cycle status of transduced cells grown in the presence or absence of IL-3. No obvious difference in cell cycle characteristics was seen in the presence of IL-3 (data not shown). However, 1 day after IL-3 deprivation a sub-G1 population appeared. As shown in Figure 2B, the percentage of sub-G1 cells was significantly reduced in BaF3 cells over-expressing murine BCL2A1a or human BCL2A1 (20% and 5%, respectively) when compared with control cells (MOCK and vector both 45%). Sub-G1 cells are usually associated with apoptosis, hence we directly assessed the apoptotic status of these cells.

Cells over-expressing murine BCL2A1a or human BCL2A1 were protected against apoptosis compared to MOCK or control GFP vector-transduced cells following IL-3 deprivation. Two days following removal of IL-3, almost all MOCK and vector control cells were apoptotic, compared to 50% and 20% for murine HA-BCL2A1a and human HA-BCL2A1 (p<0.0001) (Figure 2C). Similar results were obtained in transduced murine 32Dcl3 cells deprived of IL-3, or human UT7 UT7/Epo-S1 cells deprived of erythropoietin (data not shown). These results confirm the anti-apoptotic function of BCL2A1 over-expressed in hematopoietic cells using lentiviral vectors.

### BCL2A1a Over-expression Increases the Engraftment of Hematopoietic Stem Cells

**Figure 3:**
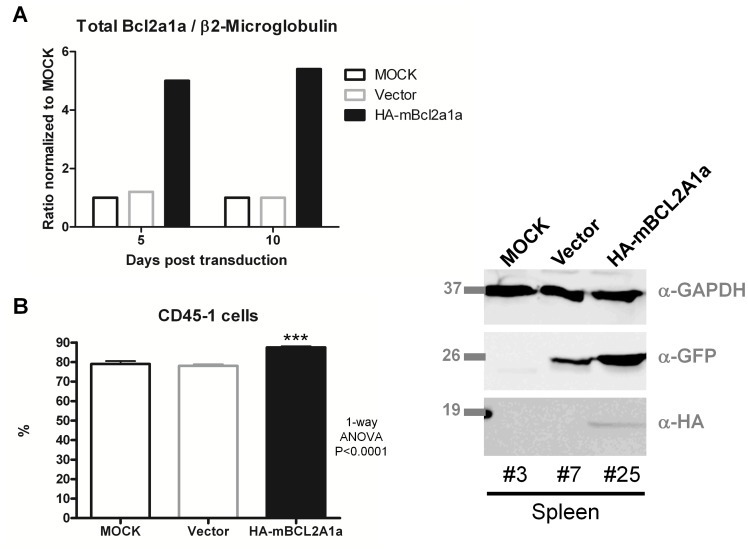
Expression of Bcl2a1a transgene in murine HSPCs and engraftment in primary recipient mice. (A) Ratio of mRNA expression of total *Bcl2a1a* over β2-Microglobulin in transduced lineage negative cells from bone marrow cells 5 or 10 days post-transduction. The MOCK ratio was set up to 1 and ratios for Vector and HA-mBcl2a1a samples were normalized to MOCK. Primers for *Bcl2a1a* did not discriminate endogenous and exogenous mRNAs. (B) Percent engraftment of donor cells (Ly5.1) was assessed in the peripheral blood leukocytes of recipient mice (Ly5.2) one month post-transplant. (C) Expression of BCL2A1a protein was assessed in the spleens of MOCK mouse #3, vector mouse #7, and BCL2A1a mouse #25 via western blot. Data averages plus standard deviations were plotted. Antibodies used as for Figure 1.

We studied the impact of over-expression of murine BCL2A1a on the behavior of primary hematopoietic stem and progenitor cells using a murine congenic transplantation model. Bone marrow cells from Ly5.1 C57BL/6 mice were transduced with lentiviruses expressing GFP alone or GFP/HA-BCL2A1a prior to reinfusion into sublethally-irradiated Ly5.2 C57BL/6 recipient mice. Five mice received untransduced donor cells, 15 mice received GFP-vector transduced cells, and 15 received GFP-murine BCL2A1a-vector transduced donor cells. From the donor cells kept in vitro we determined the fold increase expression at the mRNA level of *Bcl2a1a* to be 5 compared to controls (MOCK and Vector) after 5 or 10 days post-transduction (Figure 3A). The set of primers did not discriminate between endogenous and exogenous murine *Bcl2a1a*, but primers designed to detect the HA-tag only showed expression of exogenous *Bcl2a1a* in cells transduced with the HA-*Bcl2a1a* construct (data not shown). Mice receiving bone marrow cells over-expressing HA-BCL2A1a had significantly better engraftment than mice receiving MOCK or vector control cells (p<0.0001) (Figure 3B). The level of peripheral blood cells expressing GFP was also higher in the Bcl2A1a-GFP versus GFP alone recipients, 80% versus 10% respectively. We confirmed the in vivo expression of HA-BCL2A1a in primary hematopoietic cells by western blot of the spleen, as shown in Figure 3C.

### Mice Over-expressing BCL2A1a Develop Aberrant Hematopoiesis, Organomegaly and Tumor Masses

**Figure 4:**
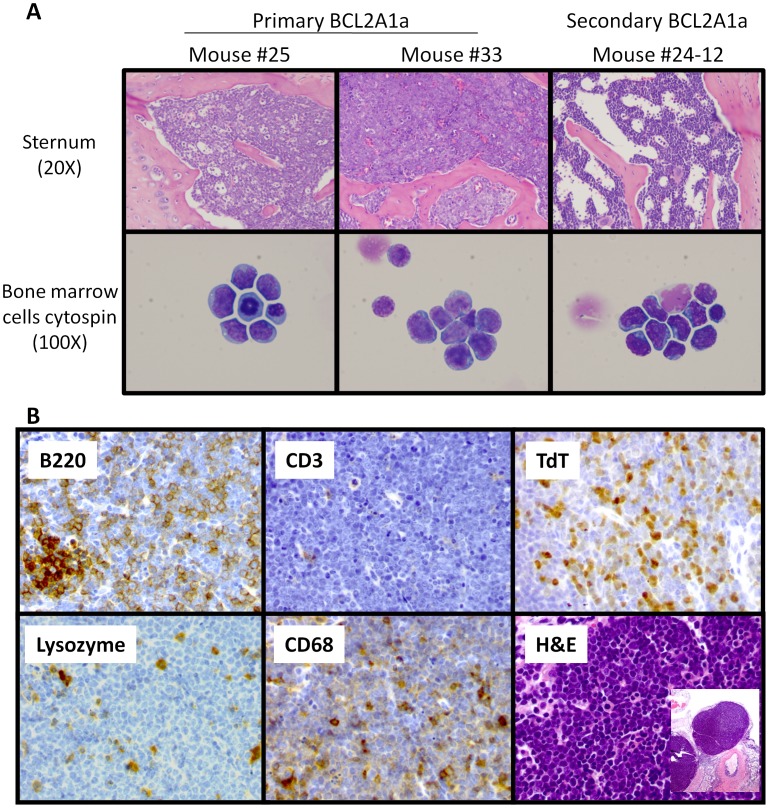
Analyses of bone marrow cells by microscopy after H&E staining and immunohistochemistry of tumor cells. (A) Micrographs obtained from H&E stained slides of sternal sections or cytospins from bone marrow cells. Primary BCL2A1a mice #25 and #33 as well as secondary mouse #24-12 are represented. Sternal sections were appreciated at 20X magnification whereas blastic cells from cytospins were appreciated at 100X magnification. (B) Example of immunohistochemical staining of lymph nodes from primary BCL2A1a mouse (#33). Lymph node sections were stained with antibodies directed against antigens including B220 for B-cells, CD3 for T-cells, TdT for immature leukemic cells, lysozyme for myeloid cells, CD68 for monocytes or histiocytes. Sections were also stained with Hematoxylin and Eosin (H&E).

**Figure 5:**
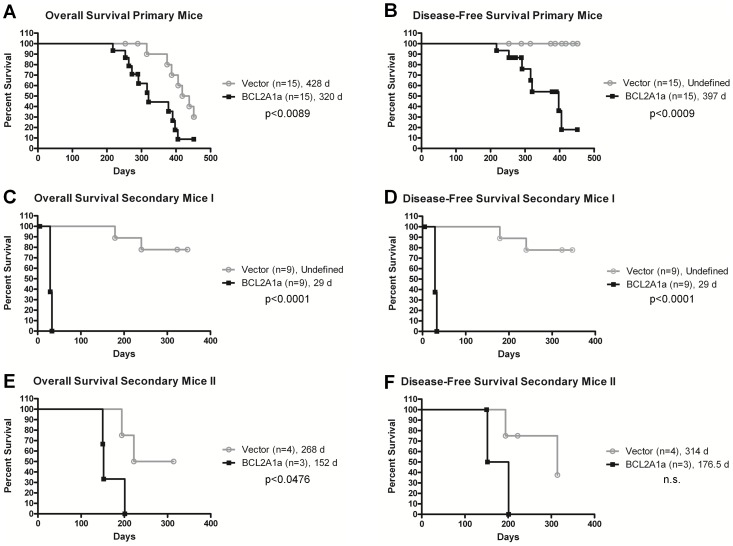
Survival curves. Kaplan-Meier overall survival and disease-free survival curves for primary transplanted mice and secondary transplanted mice (vector control or BCL2A1a): Overall survival includes deaths due to hematologic tumors and all other causes (radiation toxicity and related premature aging), with censoring of primary animals only when euthanized and utilized for secondary transplants. Overall survival curves shown in (A), (C), and (E) respectively correspond to primary mice, first set of secondary mice, and second set of secondary mice. The disease-free survival curves show development of hematologic disease (leukemia/lymphoma) as assessed by autopsy and histopathology analyses of all organs. Animals that died of non-hematologic causes were censored at their time of death. Disease-free survival curves shown in (B), (D), and (F) respectively correspond to primary mice, first set of secondary mice, and second set of secondary mice. The number of mice constituting a group is indicated between parenthesis, and median survivals are indicated in days. n.s. = not significant.

Up to six months following transplantation, all mice remained healthy and with normal blood counts. However, following this time point, mice in the BCL2A1a cohort began to show symptoms of a systemic disease with weight loss, hunching, and lack of grooming, and consequently had to be euthanized. Peripheral blood counts were still in the normal range at that time (Supplemental Figure S1A, S1B, and S1C). At postmortem, affected BCL2A1a mice, compared to GFP mice, had enlarged spleens and livers, as well as large inguinal and axillary lymph nodes (Supplemental Figure S2). In histopathological analyses multiple tissues were infiltrated with large immature blasts with a high nuclear to cytoplasmic ratio (Figure 4A). The normal architecture of bone marrow, spleen, and lymph nodes was completely effaced by these abnormal cells (Figure 4A and B). These abnormal cell populations were not seen in MOCK and vector control mice. The abnormal cells were GFP positive. Only mice receiving cells transduced with the BCL2A1a construct developed the malignant disease as shown in overall and disease-free survival analyses (Figure 5A and B). We did not observe this type of malignant phenotype in the GFP vector or MOCK mice (Figure 5B).

We further characterized the blast cells infiltrating tissues in BCL2A1a mice, because they were not consistently positive for standard lineage markers used for flow cytometric characterization of murine hematopoietic cells in peripheral blood and bone marrow (Supplemental Figure S3). Immunohistochemical staining (Figure 4B) were performed in 8 primary mice: 1 MOCK control (#3), 1 vector control (#7), and 6 primary BCL2A1a mice (#24, 25, 28, 29, 32, and 33). The 2 control mice and 1 primary BCL2A1a mouse #24, which was healthy but used for secondary transplant, had unremarkable morphology and no evidence of abnormal tissue infiltration as demonstrated by immunohistochemical staining. Three primary mice had morphologic evidence of an extensively disseminated hematologic malignancy involving spleen, bone marrow and lymph nodes (#25, 28, and 33). Tumor cells were positive for the B-cell marker B220 and the immature leukemic marker TdT, and were negative for myeloid and monocytic markers (MPO, Lysozyme, CD68, Mac2), T-cell antigen (CD3), and mature germinal center-derived B cell antigen (peanut agglutinin), consistent with the diagnosis of B-lymphoblastic leukemia/lymphoma. For 2 primary BCL2A1a mice (#32, 29) the animals showed neoplastic cells positive for TdT but negative for all other markers, including B220, consistent with the diagnosis of an acute undifferentiated leukemia.

**Figure 6:**
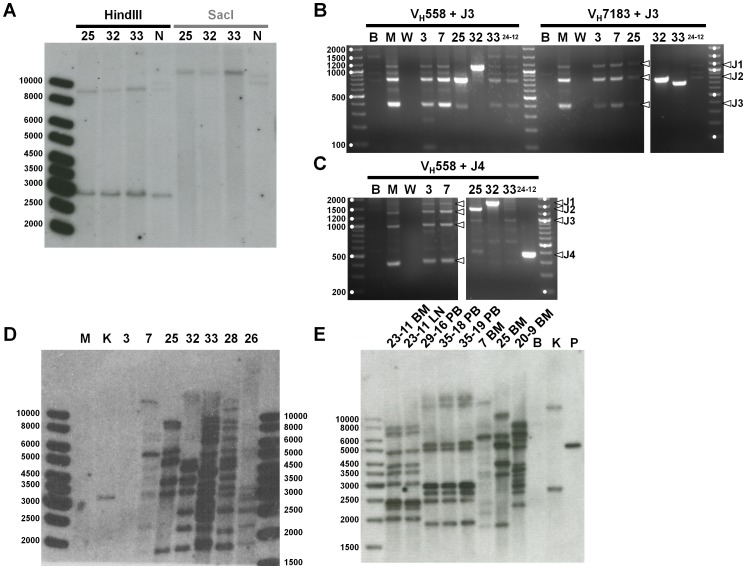
Lineage Analyses and clonality of Tumor Cells. (A) T-cell receptor β gene rearrangement. Genomic DNA isolated from bone marrow of three primary mice diagnosed with leukemia/lymphoma (#25, 32, and 33) and expressing HA-tagged BCL2A1a were subjected to a Southern blot analysis using a Cβ2 probe able to recognize the Cβ1 and Cβ2 region of the T-cell receptor β gene. Germline genomic DNA should produce bands of 8.9 and 2.9 Kb when digested with HindIII, and a band of 11.1 Kb when digested with SacI. NIH3T3 cells (N) were used as a control. Ladder size is given in base pairs. (B, C) Immunoglobulin gene rearrangements. PCRs to assess V to DJ rearrangements were performed with primers identifying the degenerated heavy variable region (V_H_558 and V_H_7183) as forward primers and J3 (B) or J4 (C) primers recognizing J3 or J4 genes. PCRs were performed on genomic DNA isolated from the bone marrow of control mice (MOCK #3 and vector #7), three primary mice diagnosed with leukemia/lymphoma (#25, 32, and 33), and one secondary mouse also diagnosed with leukemia (#24-12 expressing HA-tagged BCL2A1a). Genomic DNA obtained from BaF3 cells (B) and regular bone marrow from C57BL/6 mouse (M) were used as controls as well as a no-template control with water (W). Ladder size is given in base pairs, and are designated by a white dot. White arrows indicate the J gene involved in the rearrangement. The complete panel of PCRs for V to DJ and V to J rearrangements as well as germline configurations are shown in Supplemental Figure S4. (D) Southern blot performed with a GFP probe on genomic DNA isolated from whole bone marrow cells of primary mice. Genomic DNA obtained from regular bone marrow from C57BL/6 mouse (M) and from K562/D33 (K) harboring one copy of pRRL.PPT.SF.IRES.GFPpre/HA were respectively used as negative and positive controls. Control mice MOCK #3 and vector #7 were also used as controls. #25, 32, 33, 28, and 26 were primary mice diagnosed with leukemia/lymphoma. Ladder size is given in base pairs. (E) Southern blot performed with GFP probe on genomic DNA isolated from whole bone marrow (BM), lymph node (LN), or peripheral blood (PB) cells of secondary mice. Genomic DNA obtained from BaF3 cells (B) and from K562/AL (K) harboring two copy of GFP vector were respectively used as negative and positive controls. Plasmid pRRL.PPT.SF.IRES.GFPpre/HA-BCL2A1a was also used as a positive control. Mice vector #7 and primary BCL2A1a #25 were used as controls. Secondary BCL2A1a #29-16, 35-18, 35-19, and vector #20-9 mice belong to the first set of secondary transplant. Secondary BCL2A1a mouse #23-11 belongs to the second set of secondary transplant. All secondary mice were diagnosed with lymphoma/leukemia. Ladder size is given in base pairs.

We analyzed the tumor cells at the molecular level. The possibility of T cell lineage of the blast cells was ruled out via Southern blot, which demonstrated germline configuration of the TCR genes (Figure 6A). The B cell origin of the malignant cells was confirmed at the molecular level using a PCR-based method adaptated from Schlissel and colleagues [28]. Bone marrow infiltrated with tumor from affected mice had clonal V to DJ rearrangements of the immunoglobulin variable heavy chain gene. For instance mice #25, 32, and 33 clearly show only one amplicon when the VH558 and/or VH7183 primer was used with J3 or J4 primers (Figure 6B and C). Control mice represented by MOCK mouse #3 and vector mouse #7 showed polyclonal populations comparable to a non transplanted C57BL/6 mouse (M). (Supplemental Figure S4). Together these results showed that the malignancy is a lymphoma/leukemia of B-cell origin.

Southern blot performed with a GFP probe on whole bone marrow cells showed that primary BCL2A1a mice diagnosed with the lymphoid disease (#25, 26, 28, 32, and 33) did not share the same profile of integration, suggesting that different infiltrating clones were responsible for the disease (Figure 6D).

### The Malignant Disease is Transplantable

In order to further study the behavior of BCL2A1a-transduced cells, we performed secondary transplants consisting of transplanting marrow from primary mice engrafted 6 months or more with transduced cells into secondary recipients. We chose a ratio of 1 primary for 3 secondary mice, and reinfused the cells in this experiment into non-irradiated secondary mice. All mice receiving cells from BCL2A1a primary mice died within a month post transplant, with a disease phenotype similar to the one described above for primary mice (Figure 5C and 4A). Immunohistochemistry was only positive for the Tdt marker of blastic cells (data not shown), but the PCR was positive for B-cell rearrangement using the J4 primer (Figure 6C) documenting the B-cell origin of the infiltrating cells. Of note, secondary mice receiving BCL2A1a-transduced primary marrow developed the phenotype rapidly, even when the primary donors showed no disease manifestation at the time of transplant. BCL2A1a secondary mice had leukemic peripheral blood (Supplemental Figure S1D, S1E, and S1F). There was a significant difference in the survival of secondary mice receiving BCL2A1a versus GFP vector primary cells. The mean engraftment assessed by the presence of Ly5.1 cells in the peripheral blood was of 84.3% for BCL2A1a secondary mice whereas for the vector control it was only 2.5%. All the BCL2A1a secondary mice were diagnosed with the malignant disease (Figure 5D). Two of the vector control mice established from the same primary mouse (#20) also developed a hematologic malignancy late after transplantation, with a lymphomatous phenotype (Figure 5C and 5D), potentially related to insertional mutagenesis. For these cases donor cells (Ly5.1) went up from 1.5% to 74.1% in the peripheral blood at the time of death with 96% GFP positivity.

In our second attempt secondary mice were sublethally irradiated at 900 rads. We observed a significant difference in the overall survival between BCL2A1a and vector control mice (Figure 5E), however due to a reduced cohort size, the disease-free survival was not significantly different (Figure 5F). All the BCL2A1a mice developed a comparable malignant disease.

Southern blot with GFP probe showed different profiles of integration for BCL2A1a secondary mice from the first cohort (#29-16, 35-18, 35-19) and the second cohort (23-13) suggesting different clones responsible for the disease (Figure 6E). We can also see that infiltrating cells tested from different organs show the same profile (e.g. 23-13 BM and 23-13 LN) (Figure 6E).

## Discussion

We recently reported an adverse event in a monkey treated with genetically modified stem cells expressing GFP as a marker from a MSCV-derived retrovirus [2]. After a remarkable *in vivo* clonal expansion of the myeloid progeny derived from a single transduced hematopoietic progenitor with an insertion in the *BCL2A1* locus, accompanied by normal blood counts and myeloid differentiation early after transplantation, the animal eventually developed AML derived from the same clone, and died six years post-transplant [1]. The tumor cells over-expressed *BCL2A1* mRNA. Prior to our study, a link between BCL2A1 and AML has only been suggested [23]. Leukemias and clonal dominance in both animal models and human gene therapy clinical trials have been previously linked to insertional activation of *LMO2*, the *MDS1/EVI1* gene complex, *HOXB4*, and a number of other transcription factor genes, but not previously to activation of an anti-apoptotic gene [30]–[33].

To address whether BCL2A1 over-expression can promote development of leukemia/lymphoma, in the absence of a specific cooperating oncogene such as MYC, we used a murine bone marrow transplantation model. We used a lentiviral vector expressing HA tagged BCL2A1a along with a GFP marker to over-express this protein in hematopoietic stem and progenitor cells that were then reinfused into congenic mice. While any integrating vector can potentially induce leukemia by insertional mutagenesis alone, the risk is relatively low using lentiviral as compared to standard murine retroviral vectors, and comparison to the control GFP vector allows analysis of the impact of BCL2A1a over-expression independent of any vector effects [34]. Analysis of insertion sites from the bone marrow of two BCL2A1a mice (primary #25 and secondary #23-11) with tumors, and one vector control mouse without tumor (primary #7) did not reveal vector insertions within or adjacent to proto-oncogenes such as Myc. None of the insertion sites were shared between independent tumors (Supplementary Table S1). We confirmed that murine BCL2A1a and human BCL2A1 over-expressed via this lentiviral vector could protect several hematopoietic cell lines from apoptosis in vitro. Following transplantation, murine marrow cells transduced with the BCL2A1a vector demonstrated higher engraftment levels compared to cells transduced with the control vector, possibly resulting from the anti-apoptotic function of BCL2A1a, allowing better survival of successfully-transduced cells in vitro during culture, and in vivo during the process of homing and engraftment. For the first six months, hematopoiesis in these mice appeared normal and was analogous to the initial clonal dominance with normal hematopoiesis observed in our primate model when the *BCL2A1* gene was activated by vector insertion [1]. With longer follow-up, we found that BCL2A1a over-expression resulted in a malignant hematologic disease with prolonged latency.

Our previous study [2] and the study from Beverly and Varmus [24], led us to hypothesize that BCL2A1a would induce a myeloid leukemia. However, we characterized the malignant tumors in individual mice as lymphoblastic leukemias/lymphomas of the B-cell lineage. The discrepancy between our findings and the previous murine study [24] can be explained by the fact that in their experiments BCL2A1 was over-expressed along with MYC, whereas we over-expressed BCL2A1a alone. It has been established that MYC alone induces a myeloid leukemia [35], and the co-expression of high levels of all anti-apoptotic proteins in their model simply accelerated the time to presentation of the myeloid leukemia phenotype. Even oncogenes with a very clear link to primarily myeloid malignancies in humans, such as *BCR-ABL*, can result primarily in a lymphoid malignant phenotype when over-expressed in murine hematopoietic progenitors [36]. The disease induced by BCL2A1a was more aggressive and had much shorter latency in secondary recipients. The phenotype was transplantable and of primitive origin. It was interesting to note that the phenotype was more aggressive and the latency shorter in non-ablated secondary recipients. This initially surprising finding is actually in accordance with the work of Sadat and colleagues [37]. Retrovirally-induced leukemias and clonal dominance in their murine model was more rapid and frequent in sub-lethally-irradiated as compared to irradiated recipients.

In conclusion, we determined that the over-expression of BCL2A1a in murine hematopoietic stem and progenitor cells can prevent apoptosis, enhance engraftment, and promote eventual outgrowth of fully transformed leukemic cells, primarily of the B lymphoid lineage. Our studies suggest that further investigation of BCL2A1 as a participant in hematologic transformation is indicated, and it adds *BCL2A1* to the list of proto-oncogenes that can be activated by insertional mutagenesis.

## Supporting Information

Figure S1 **Blood cell count at the time of sacrifice.** Blood cell counts at the time of sacrifice were determined and compared by a t-test for vector control and BCL2A1a groups. (A, B, C) respectively represent the primary mice cohort for WBC, RBC, and platelets. (D, E, F) respectively represent the first secondary mice cohort for WBC, RBC, and platelets. Data averages plus standard deviations were plotted. WBC  =  white blood cells, RBC  =  red blood cells, k/uL  =  thousands of cells per microliter, M/uL  =  millions of cells per microliter, n.s.  =  not significant.
(TIF)

Figure S2 **BCL2A1a Recipient Mice Development of Lymphadenopathy and Organomegaly.** (A) Representative pictures of each group of recipient mice (#3 =  MOCK, #7 =  vector, and #25 =  BCL2A1a). Arrows indicate lymph nodes (blue), spleen (green), and liver (red). (B,C) The weights of the spleen and liver at sacrifice were determined and compared. For primary mice, the 3 groups were compared using one way ANOVA test for MOCK, vector, and BCL2A1a mice. (D,E,F,G) For secondary mice, vector and BCL2A1a mice were compared using an unpaired t-test. (D) and (E) respectively show the weight of liver and spleen obtained for the first set of secondary transplanted mice. (F) and (G) respectively show the weight of liver and spleen obtained for the second set of secondary transplanted mice. Data averages plus standard deviations were plotted. mg  =  milligrams, n.s.  =  not significant.
(TIF)

Figure S3 **Analyses of peripheral blood and bone marrow cells by flow cytometry at the time of sacrifice.** Peripheral blood or flushed bone marrow cells were stained with antibodies recognizing Ly5.1 and Ly5.2 for chimerism, as well as antibodies recognizing CD3e, B220, and CD11b to respectively detect T-Cells, B-Cells, and granulocytes (See Material and Methods section). (A, C, E, G) represent data obtained for the peripheral blood of vector control mouse #7, primary BCL2A1a mice #25 and #33, and secondary BCL2A1a mouse #24-12. (B, D, F) represent data obtained for the bone marrow of vector control mouse #7 and primary BCL2A1a mice #25 and #33.
(TIF)

Figure S4 **Analyses of immunoglobulin heavy variable chain rearrangement.** All PCRs were carried out on genomic DNA isolated from bone marrow with either J3 (A) or J4 (B, C) as reverse primers with a unique sequence. Forward primers were used to assess germline configuration, V to DJ, or D to J rearrangements. Germline configuration was assessed with Mu0 primer. V to DJ rearrangements were assessed with degenerated primers that identified heavy variable regions (V_H_558, V_H_7183, and V_H_Q52). D to J rearrangements were assessed with degenerated primers D_H_L and D_H_R identifying D genes. Primers were described by Schlissel and colleagues [28]. Mice tested and controls are the same than for Figure 6. B  =  BaF3 cells, M  =  regular bone marrow from C57BL/6 mouse, and W  =  water. Ladder size is given in base pair and are emphasized by a white dot. White arrows indicate the J gene involved in the rearrangement. Black arrow indicates germline configuration.
(TIF)

Table S1 **Analyses of lentiviral insertion sites.** Mapping of lentiviral insertion sites from the bone marrow cells of two BCL2A1a mice (primary #25 and secondary #23-11) with tumors, and one vector control mouse without tumor (primary #7). Mice #7 and #25 were analyzed by LAM-PCR [38] and mouse #23-11 was analyzed by LM-PCR [39]–[40].
(XLSX)
