# Supplementary figures and images for: BCL2A1a Over-Expression in Murine Hematopoietic Stem and Progenitor Cells Decreases Apoptosis and Results in Hematopoietic Transformation
Source: PLoS One. 2012 Oct 30;7(10):e48267. doi: 10.1371/journal.pone.0048267 (PMC3484072; doi:10.1371/journal.pone.0048267)

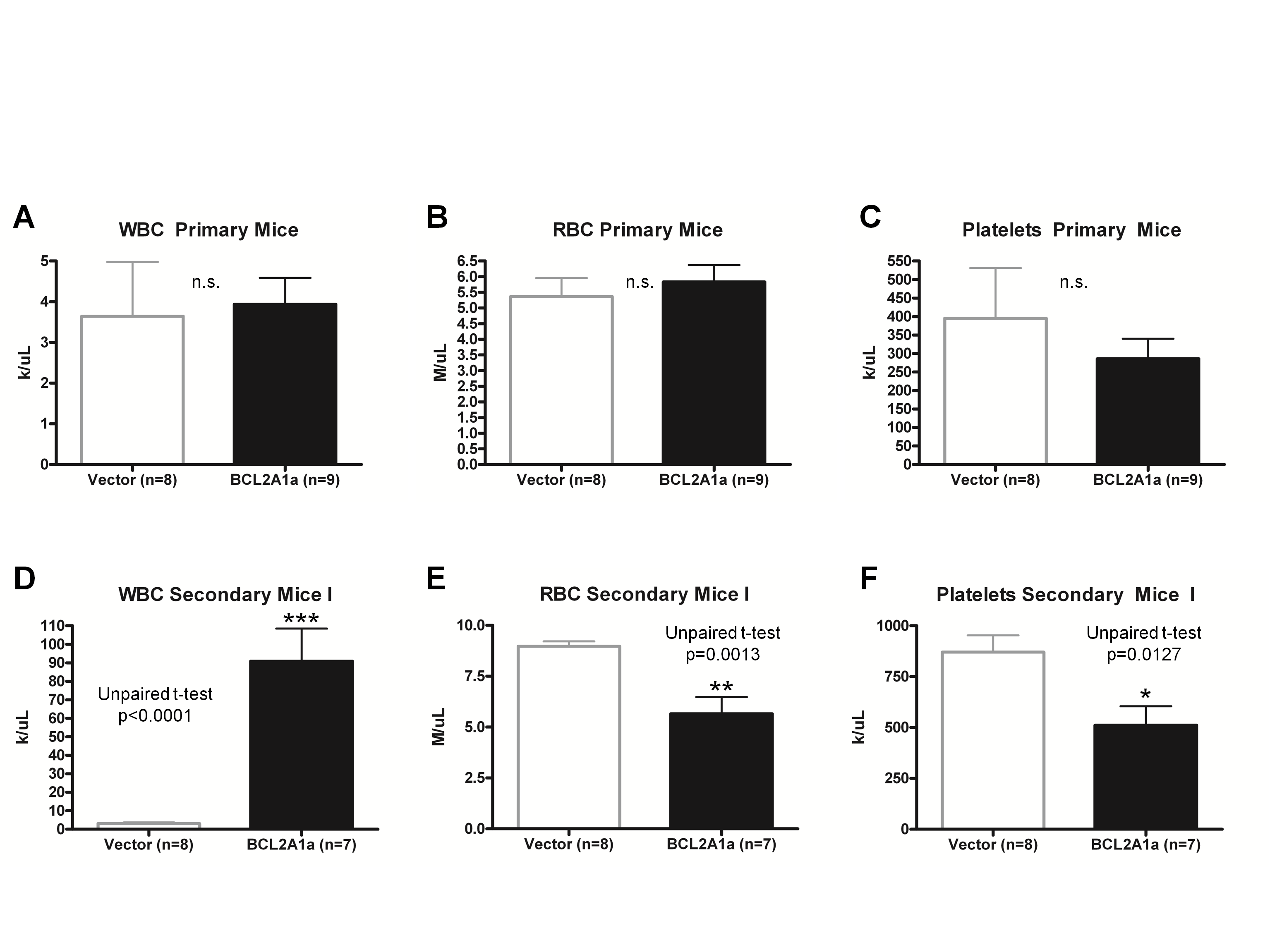

Supplement: Figure S1 — Blood cell count at the time of sacrifice. Blood cell counts at the time of sacrifice were determined and compared by a t-test for vector control and BCL2A1a groups. (A, B, C) respectively represent the primary mice cohort for WBC, RBC, and platelets. (D, E, F) respectively represent the first secondary mice cohort for WBC, RBC, and platelets. Data averages plus standard deviations were plotted. WBC = white blood cells, RBC = red blood cells, k/uL = thousands of cells per microliter, M/uL = millions of cells per microliter, n.s. = not significant. (TIF) [file pone.0048267.s001.tif]

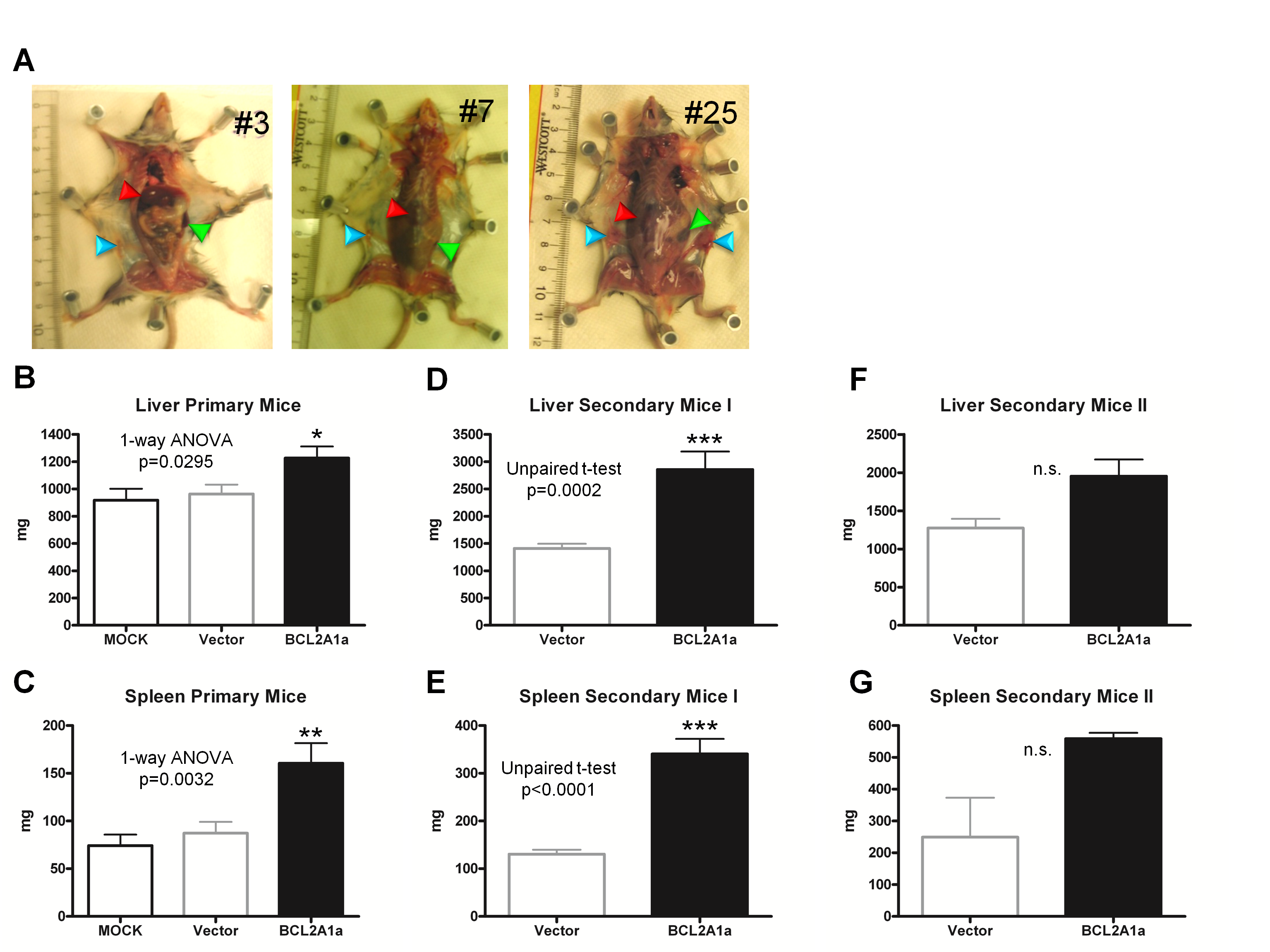

Supplement: Figure S2 — BCL2A1a Recipient Mice Development of Lymphadenopathy and Organomegaly. (A) Representative pictures of each group of recipient mice (#3 = MOCK, #7 = vector, and #25 = BCL2A1a). Arrows indicate lymph nodes (blue), spleen (green), and liver (red). (B,C) The weights of the spleen and liver at sacrifice were determined and compared. For primary mice, the 3 groups were compared using one way ANOVA test for MOCK, vector, and BCL2A1a mice. (D,E,F,G) For secondary mice, vector and BCL2A1a mice were compared using an unpaired t-test. (D) and (E) respectively show the weight of liver and spleen obtained for the first set of secondary transplanted mice. (F) and (G) respectively show the weight of liver and spleen obtained for the second set of secondary transplanted mice. Data averages plus standard deviations were plotted. mg = milligrams, n.s. = not significant. (TIF) [file pone.0048267.s002.tif]

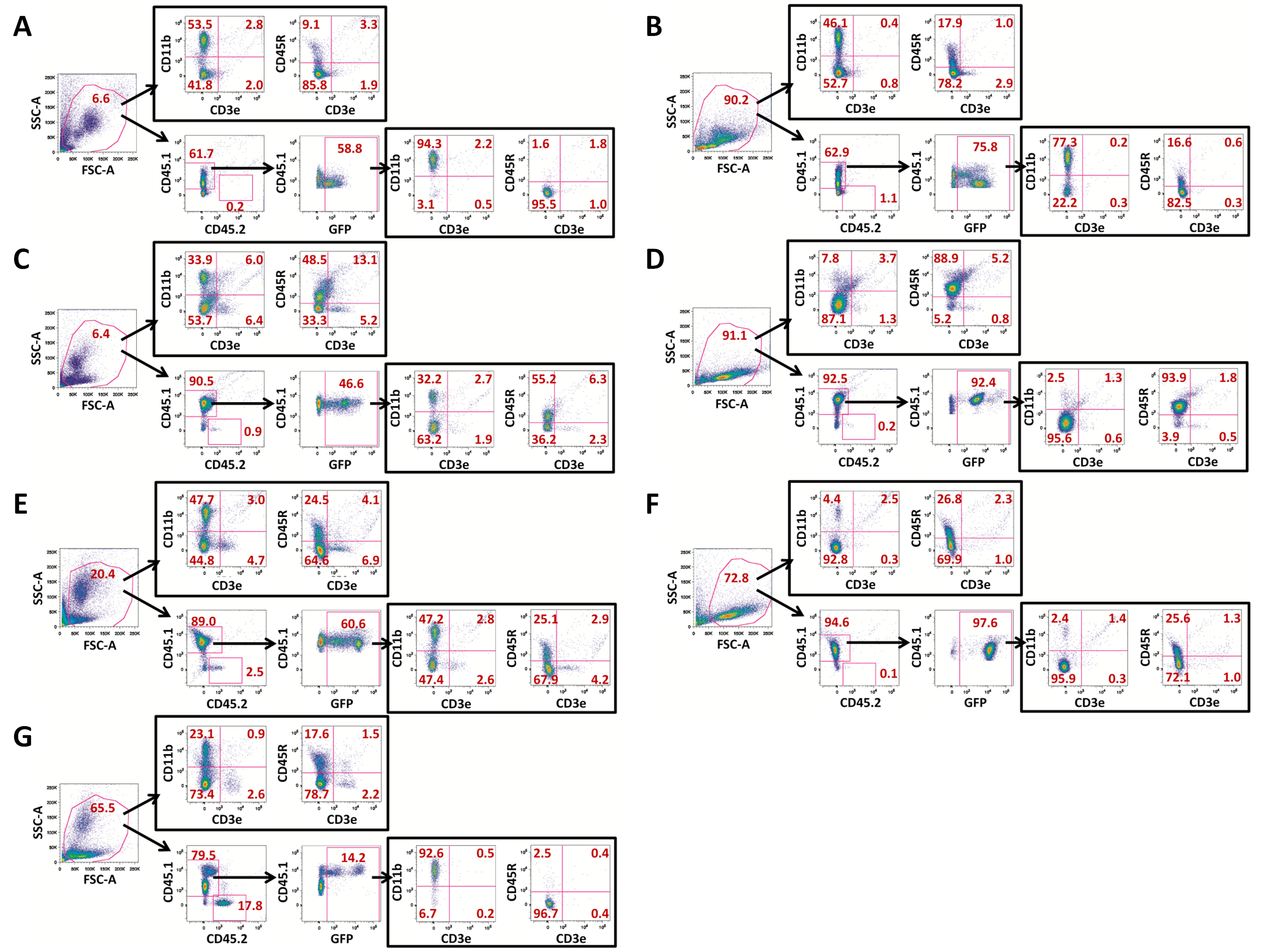

Supplement: Figure S3 — Analyses of peripheral blood and bone marrow cells by flow cytometry at the time of sacrifice. Peripheral blood or flushed bone marrow cells were stained with antibodies recognizing Ly5.1 and Ly5.2 for chimerism, as well as antibodies recognizing CD3e, B220, and CD11b to respectively detect T-Cells, B-Cells, and granulocytes (See Material and Methods section). (A, C, E, G) represent data obtained for the peripheral blood of vector control mouse #7, primary BCL2A1a mice #25 and #33, and secondary BCL2A1a mouse #24-12. (B, D, F) represent data obtained for the bone marrow of vector control mouse #7 and primary BCL2A1a mice #25 and #33. (TIF) [file pone.0048267.s003.tif]

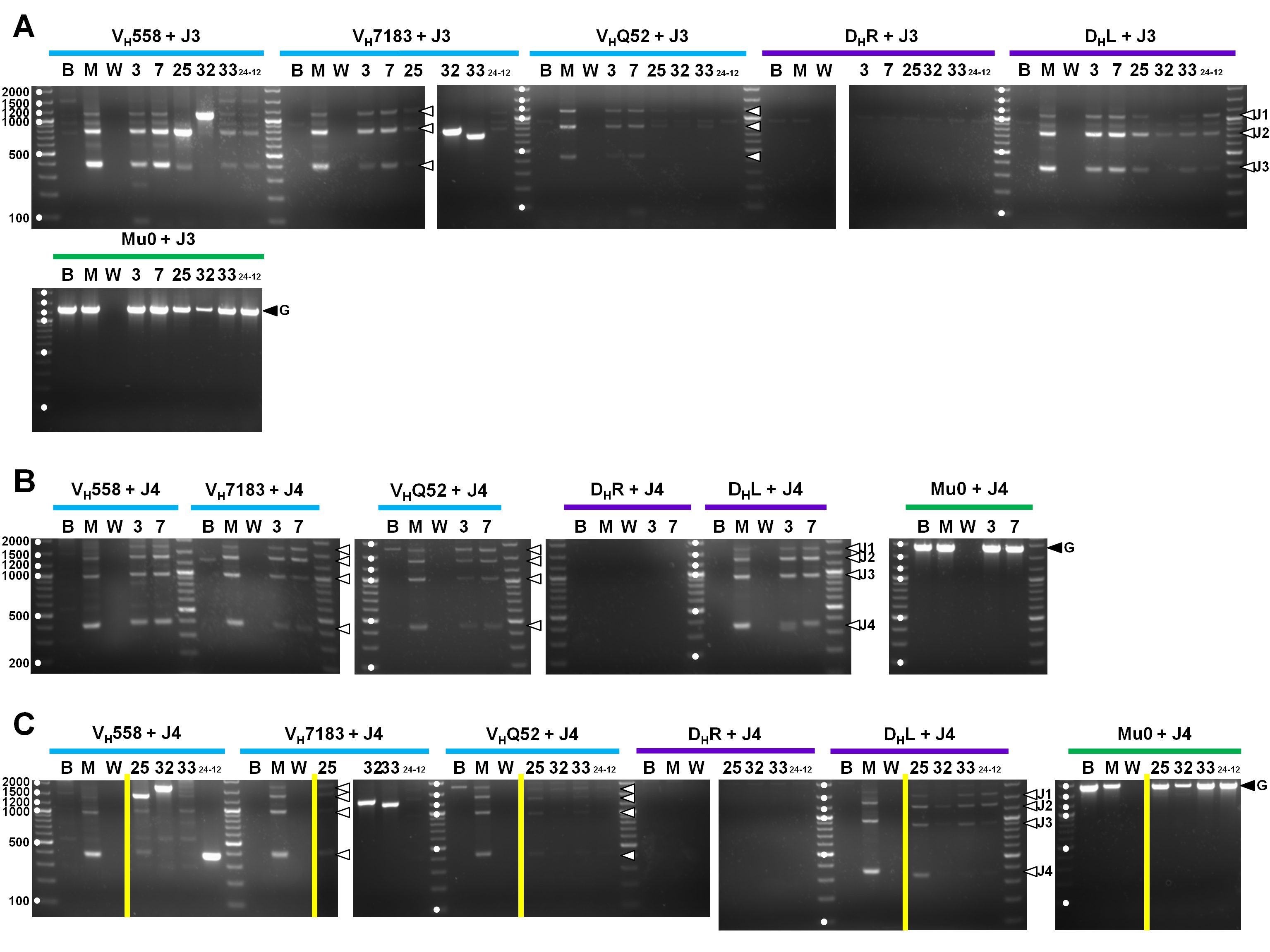

Supplement: Figure S4 — Analyses of immunoglobulin heavy variable chain rearrangement. All PCRs were carried out on genomic DNA isolated from bone marrow with either J3 (A) or J4 (B, C) as reverse primers with a unique sequence. Forward primers were used to assess germline configuration, V to DJ, or D to J rearrangements. Germline configuration was assessed with Mu0 primer. V to DJ rearrangements were assessed with degenerated primers that identified heavy variable regions (VH558, VH7183, and VHQ52). D to J rearrangements were assessed with degenerated primers DHL and DHR identifying D genes. Primers were described by Schlissel and colleagues [28]. Mice tested and controls are the same than for Figure 6. B = BaF3 cells, M = regular bone marrow from C57BL/6 mouse, and W = water. Ladder size is given in base pair and are emphasized by a white dot. White arrows indicate the J gene involved in the rearrangement. Black arrow indicates germline configuration. (TIF) [file pone.0048267.s004.tif]
